# WHAT DOES SUCCESS MEAN IN THE CONTEXT OF ELDER ABUSE INTERVENTION FROM THE PERSPECTIVE OF VICTIMS?

**DOI:** 10.1093/geroni/igz038.3173

**Published:** 2019-11-08

**Authors:** David Burnes, Jessica Hsieh, Clara Scher, Paula Zanotti, Chelsie O Burchett, Jo Anne Sirey, Mark Lachs

**Affiliations:** 1 University of Toronto, Toronto, Ontario, Canada; 2 Weill Cornell Medicine, White Plains, New York, United States; 3 Weill Cornell Medicine, New York, New York, United States; 4 Weill Cornell Medical College / New York Presbyterian Hospital, New York, New York, United States

## Abstract

Adult protective services (APS) and other community-based agencies respond to hundreds of thousands of elder abuse cases each year in the United States; however, little is known about what constitutes success in the context of elder abuse response intervention. This study explored the meaning of elder abuse intervention success from the perspective of victims themselves toward the development of a victim-centric taxonomy of outcomes. Guided by a phenomenological qualitative methodology, this study conducted in-person, semi-structured interviews with a sample of elder abuse victims (n = 30) recruited from APS in the states of Maine, New York, and California, as well as a community-based elder abuse social service program in New York City. To enhance trustworthiness, two researchers independently analyzed transcript data to identify key transcript statements into themes. Outcomes of success were identified across broad domains related to the victim, perpetrator, victim-perpetrator relationship, family system, and home environment. Specifically, common themes represented outcomes related to victim safety, autonomy, social support, and state of mind; perpetrator independence and accountability; and victim-perpetrator separation. For decades, the field of elder abuse has struggled to understand how to define success in the context of community-based intervention from a client-centered perspective. The taxonomy developed in this study provides a comprehensive and conceptually organized range of successful outcomes to serve as infrastructure for the development of meaningful intervention outcome measures. This study represents one of the largest efforts to understand and integrate the perspectives and needs of victims into elder abuse intervention practice/research to date.

